# Spatial Detection of Pb in Life Sciences: Advances and Limitations

**DOI:** 10.1021/acsomega.5c08427

**Published:** 2025-11-14

**Authors:** Conor J. Dixon, Paul D. Morton

**Affiliations:** † School of Neuroscience, 1757 Virginia Polytechnic Institute and State University, Blacksburg, Virginia 24061, United States; ‡ Department of Biomedical Sciences and Pathobiology, Virginia-Maryland College of Veterinary Medicine, Virginia Polytechnic Institute and State University, Blacksburg, Virginia 24061, United States

## Abstract

Lead (Pb) is a significant worldwide environmental contaminant. Elevated blood-lead levels during childhood can affect development and are associated with cognitive impairments, learning disabilities, and attentional deficits later in life. Clinical and toxicological assessments of Pb exposure are generally limited to blood lead level (BLL) tests. BLLs are a transient measure of Pb, as Pb itself distributes and deposits all throughout the body, including all major organs, bones, and the central nervous system. Understanding how BLLs relate to systemic and cellular Pb uptake is crucial for guiding treatments and therapies, yet tissue biopsies of the nervous system in otherwise healthy patients are often infeasible. Researchers have access to controlled experimental models to determine causal actions of Pb but are currently faced with limited options and techniques due to access and cost. While there have been many advances in spatial Pb detection since the early 1900s, they have not always translated to biological sciences as naturally as other research areas, such as geology or material sciences. We propose this is largely because of sample preparation, sample size, and imaging parameters (e.g., depth, scanning area, etc.). There is an urgent need for awareness of this gap in technology and the utility it will play in advancing our knowledge of Pb-induced health conditions. In this review, we discuss the various methods used to spatially detect and visualize Pb within biological samples, with special emphasis on the lack of tractable Pb detection techniques capable of generating spatial information in biological samples. We also discuss modern developments and advancements, emerging techniques in Pb detection, and suggested applications for future research endeavors.

## Graphical abstract

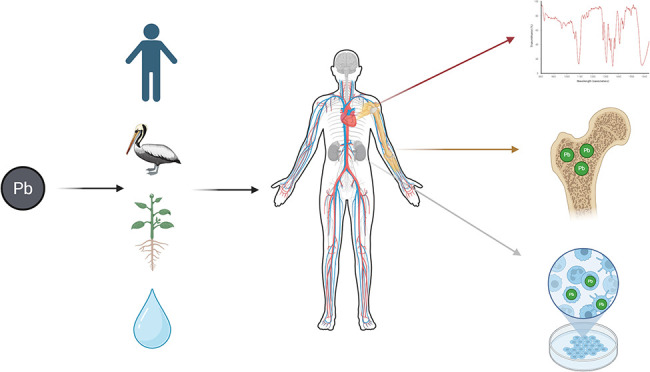

## Introduction

Lead (Pb) is a ubiquitous toxic heavy metal that has been used by humans for millennia. Ancient romans used Pb in plumbing, drinkware, and cutlery.^1^ More recently, Pb has been used in gasoline in the form of tetraethyllead.^2^ While leaded gasoline was eventually phased out in the US during the 1970s, Pb is still found in the US from a variety of sources. There are 9.2 million active Pb service lines within the US and drinking water represents the most common form of exposure; some of the most populous states within the US have the largest concentrations of active Pb service lines.^3,4^ Children are especially vulnerable to Pb exposure, as Pb can be found in toys, baby food, cereals, formula, and even the cinnamon ingredient in apple sauce.^5^ Susceptibility is further increased in children due to innate curiosities and behaviors, such as ingesting leaded paint chips and being close to contaminated surfaces as they learn to crawl. Approximately 500,000 children in the US, or 2.5% of all US children, have significantly high levels of Pb in their blood.^6,7^ Globally, this number is greater than three orders in magnitude at nearly 800,000,000 children with documented elevations in BLL.^8,9^ In addition to drinking water consumed by both children and adults, Pb is also found in many other sources more often experienced by adults including paint, batteries, electronic waste, soil, air pollution, and contaminated industrial sites.^10^ Regardless of the source of Pb exposure, the standard assessment is via blood tests followed by spectral detection and quantification of BLLs which is used as a comparative index across the world.

There is no safe BLL.^11^ In the US, acceptable BLLs were determined to be 50 μg per deciliter (μg/dL) in 1970. Despite growing awareness of Pb toxicity, this value was not updated until five decades later and stands at 3.5 μg/dL as of 2021.^12^ It is advised that children with over 3.5 μg/dL BLLs receive prompt treatment to mitigate the effects of Pb exposure, since BLLs above this threshold in children can spur developmental alterations and various behavioral challenges.^13^ Along with increased susceptibility to Pb exposure, children are also much less efficient at removing Pb from the body as compared to adults.^14^ Pb is generally absorbed orally or inhaled but can also be dermally absorbed.^15^ After ingestion, Pb is mainly excreted in bile and urine, but can be detected in the blood for around 30 days.^16^ However, 90% of ingested Pb, regardless of exposure route (e.g., ingested, inhaled, etc.) will accumulate in bones as it displaces calcium.^17^ Pb can remain in bones for decades, creating a potent source of endogenous Pb to further developmental complications.^18^

While blood tests are a reliable measure for rapid risk assessment, determinants of predicted physiological harm to an individual require subsequent tissue and fluid specific tests that are often infeasible in patients. Elucidating the tissue-specific effects of Pb exposure on biological systems requires cellular and molecular measurements. Understanding how Pb distributes in tissue and what cells are affected is important for predicting and determining physiological harm. There are a variety of ways that researchers can visualize how Pb distributes in the body both in soft tissue and bone, as well as plants. In this review, we provide a broad overview of various imaging techniques, with a special emphasis on Pb detection and spatial visualization (i.e., mapping) of Pb in biological samples, and we discuss the various pros and cons of each method. Although this review is focused solely on Pb, the issues and advances discussed are also applicable to other toxic heavy metals researched in biological systems. Lastly, we discuss current trends within the field and considerations in the development of new Pb-specific designer probes to foster high compatibility with an array of routine methods used across the different disciplines within the life sciences.

## Distribution of Pb in the Human Body

There is no known biological function for Pb. Pb is distributed readily in blood, soft tissue, and bone, and this accumulative effect has irreversible, long-term effects on a plethora of biological functions. Upon ingestion, Pb is quickly absorbed and enters the bloodstream through the small intestine. Within the small intestine itself, Pb damages gut microbiota (Figure 1), leading to Pb-induced dysbiosis.^19,20^ Pb enters the bloodstream by way of enterocytes (Figure 1), where it will then bind to proteins in red blood cells.^21^ By binding to these proteins, Pb not only distributes all over the body via the bloodstream but also affects red blood cells specifically. Pb inhibits the synthesis of hemoglobin, leading to anemia.^22^ Because of how readily Pb enters the bloodstream and binds to red blood cells, the cardiovascular system is vulnerable to Pb exposure. Pb exposure can lead to hypertension and arterial calcification (Figure 1), as well as increased risk of stroke and heart disease.^23^ Blood is primarily filtered by the liver, and Pb will evenly distribute across liver lobules^24^ (Figure 1). Pb exposure increases the risk of nonalcoholic fatty liver disease, as well as a decrease in overall liver function.^25^ From the bloodstream, Pb is transported to the blood brain barrier where it can readily cross (Figure 1). Due to its ability to mimic calcium ions (i.e., ion mimicry), Pb can affect many different calcium signaling proteins in the brain, altering a multitude of different processes.^26^ Children’s brains are particularly sensitive to Pb exposure while their skulls are growing quickly; Pb exposure in children is associated with behavioral alterations, attention deficits, and lower IQ.^13^

**Figure 1:**
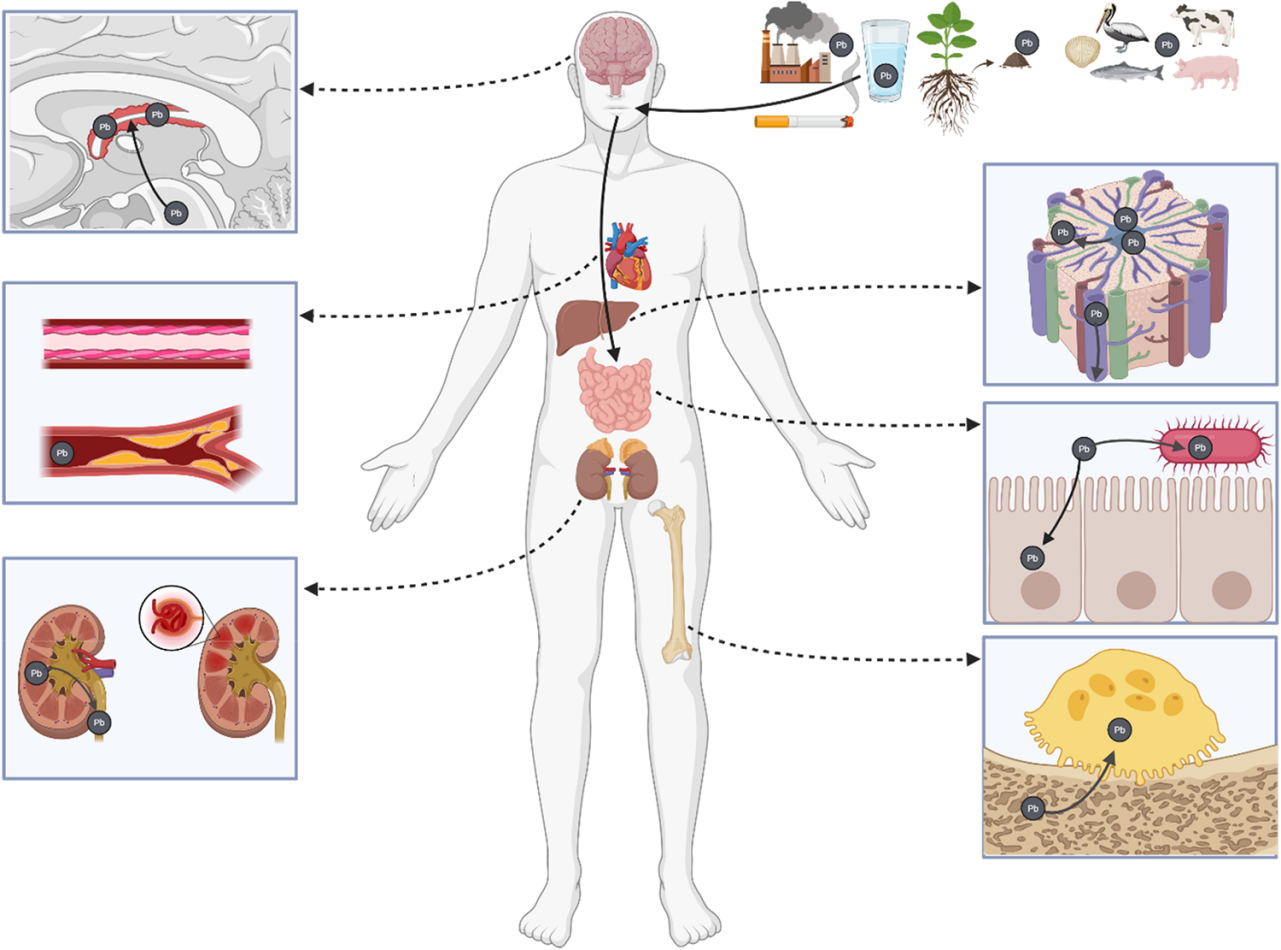
Distribution of Pb in the human body after oral ingestion. Not all affected organs are shown. Created by authors in https://BioRender.com.

Pb is primarily excreted through urine, and the kidneys are susceptible to Pb exposure (Figure 1). Pb exposure increases risks of renal dysfunction and renal toxicity.^27^ The majority of Pb that is not excreted is stored in the bones. As mentioned earlier, 90% of Pb is stored within bones, and this can lead to specific skeletal issues, such as osteoporosis and osteoarthritis.^28–30^ However, since storage in the bones acts as a source of endogenous Pb, it can affect all parts of the body for decades. Once bone is resorbed by osteoclasts (Figure 1), endogenous Pb will reenter the bloodstream. Not only can this affect the exposed persons, but it can affect reproduction as well. Pb from bones is resorbed and then delivered to the fetus via the placenta.^31^ Besides these specific systems and soft tissues mentioned, a multitude of other soft tissues and biological systems are affected by Pb exposure, such as the lungs, stomach, mitochondria and ER, to various epigenetic changes in all cell types throughout the entire body. Therefore, it is critical to evaluate and research Pb on the organ-, tissue-, cell-specific, and organelle-specific levels which require sophisticated methodologies often unavailable in research laboratories, clinics, or the field. We next broadly describe such techniques in brief beginning with a historical perspective.

### Mass Spectrometry Techniques

#### Inductively Coupled Plasma Mass Spectrometry (ICP-MS)

ICP-MS is the gold standard for detection of heavy metals in tissue. First described in 1980 and commercialized in the early 1980s (Figure 2), ICP-MS is an affordable and sensitive technique for measuring metal ions in samples.^32^ ICP-MS utilizes plasma generated from electromagnetic induction that ionizes a sample. Once these atomic ions are generated, they are extracted to the mass spectrometer, where they are separated based on their mass to charge ratio. Pb can be detected at picomolar concentrations and ICP-MS is an ideal method for studies utilizing minute amounts of Pb. Furthermore, ICP-MS is readily available at many institutions and is affordable in comparison to other methods discussed later in this review. ICP-MS is often used to detect heavy metals in bone, tissue, blood, and even soil and water. Furthermore, it is used in many different contexts, such as when researching wild animals, lab animals, farm animals, humans, and plants.^33–37^ There have been some recent advancements in ICP-MS technology, especially as it relates to single particle ICP-MS in biological samples.^38^ These kinds of advancements allow for the detection of even the smallest molar amounts of Pb in biological samples.

**Figure 2:**
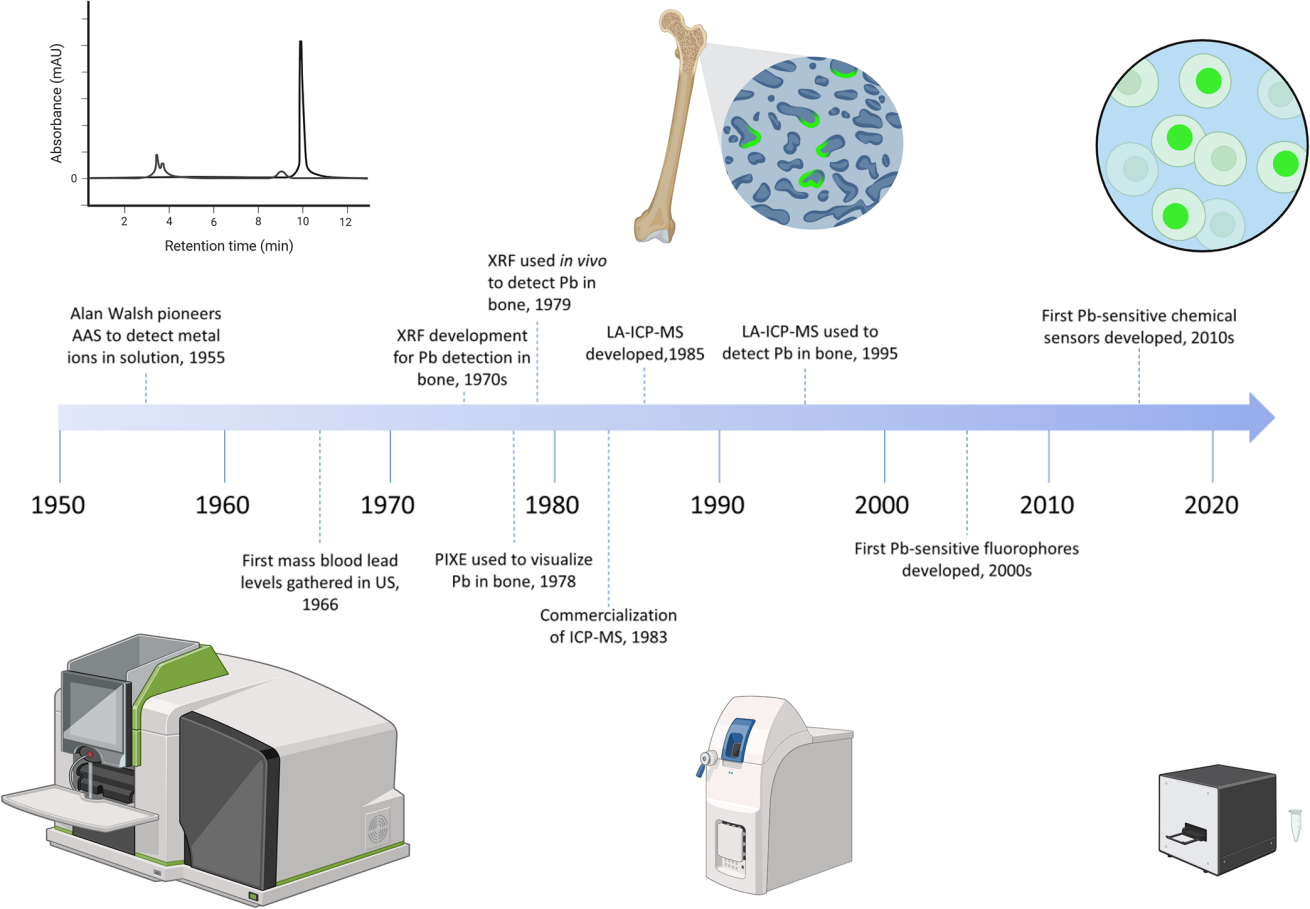
Timeline of Pb detection methods. The bottom row shows, to scale, an atomic absorption spectrometer (AAS) (bottom-left), a compact ICP-MS (bottom-center), and a UV–vis spectrometer (bottom-right). Note the decrease in size over time. The top row shows an example of an AAS spectrogram (top-left), an image from an LA-ICP-QQQ visualizing Pb deposits in bone (top-center), and a Pb-specific fluorophore within cells exposed to Pb (top-right). Note the increase in resolution over time. *References not noted in the text*: AAS, Walsh, 1966.^131^ Created by authors in https://BioRender.com.

While ICP-MS is useful in detecting Pb and other metals in a variety of biological samples, it requires the sample to be digested before introduction as a liquid to the plasma. Because of this, ICP-MS lacks spatial information that other methods can provide. ICP-MS also has a recommended amount/mass of a sample/volume of chemical digestive liquid.^32^ For certain microstructures, such as specific regions of a mouse kidney, one may need samples from several mice pooled together to achieve an adequate mass. This pooling together of samples could lead to the loss of individual data points, increasing the number of specimens/animals required to perform the study. This problem may be avoided by utilizing techniques that provide spatial visualization of the sample *in situ*. ICP-MS can be paired with several different tools to allow for the spatial detection and visualization of Pb ions in a sample.

#### Laser Ablation Inductively Coupled Plasma Mass Spectrometry (LA-ICP-MS)

LA-ICP-MS is a method that can detect various heavy metals in tissue. It is one of the most popular methods to spatially visualize Pb in soft tissue, bones, and plants. First developed in the late 1980s (Figure 2), LA-ICP-MS utilizes a laser to ablate a sample, creating fine particles.^39^ This laser ablation system is coupled to an ICP-MS, and these particles are then ionized via ICP-MS and analyzed allowing for subtle detection of heavy metal ions, such as Pb^2+^. Unlike ICP-MS alone, LA-ICP-MS can provide spatial information, both planar and depth, from the sample. Furthermore, LA-ICP-MS utilizes a solid sample and does not require digestion. However, LA-ICP-MS still destroys the sample in the process (Table 1). Regardless, due in part to the fact that LA-ICP-MS requires no digestion, it has a much higher throughput than ICP-MS alone.

**Table 1:** Comparison of Most Common Metal Detection Methods

method	sensitivity	max depth	cost	spatial	sample integrity	sample size/scan area
ICP-MS	ppt	N/A	less expensive	no	destructive (digested)	recommended 2 g/L of dissolved solid
XFM	ppb	several micrometers	expensive	yes	nondestructive	1 to 10 μm
LA-QQQ-MS	ppb	1 μm	expensive	yes	destructive (laser)	1 to 100 μm
PIXE	ppb	1 μm	very expensive	yes	nondestructive	1 μm to 1 mm

While LA-ICP-MS is relatively sensitive, the laser ablation system must be coupled to a triple quadrupole ICP-MS (LA-ICP-QQQ) to achieve nano or picomolar concentration sensitivity within a sample. LA-ICP-QQQ is utilized in many studies detecting Pb in a variety of biological samples and tissues, including liver, bone, and teeth.^24,34,40^

The ability to couple the laser ablation system to different ICP-MS technologies makes it a versatile tool. For instance, a recent study utilized a laser ablation system coupled to an octupole ICP-MS to spatially visualize Pb within rat liver, lungs, and kidney after Pb inhalation.^41^ An octupole collision/reaction cell removes more interference than a quadrupole system and potentially allows for more sensitive measurements.^42^ Compared to ICP-MS alone, LA-ICP-QQQ are expensive to purchase and to use, and they are not a common part of research infrastructure in the US. However, there have been recent advancements and improvements in the technology itself, including improved sensitivity, as well as increased spatial resolution via computational integration.^43,44^ These advancements potentially allow for the spatial visualization of even the smallest concentrations of Pb in biological samples. For an in-depth review of LA-ICP-MS and its use in the biological sciences, see *Doble et al, 2021*.^45^

### Other MS-Related Techniques

#### Secondary Ion Mass Spectrometry (SIMS)

SIMS has been employed extensively in surface analysis of inorganic materials. While the first use of SIMS was in the 1960s, it was not utilized in the life sciences until the 1980s. SIMS is unique to mass spectral imaging, in that it utilizes a primary ion source to fire a concentrated ion beam at a solid sample. This sputtering, high energy beam generates secondary ions from the surface of this sample. Secondary ions are ejected into a vacuum environment and subsequently transferred to a mass analyzer which separates and analyzes the ions. A variety of mass analyzers, such as quadrupole analyzers, time-of-flight (ToF-SIMS), or double-focusing sector field mass analyzers (NanoSIMS) can be used with SIMS, with ToF-SIMS and NanoSIMS ranking among the most common. NanoSIMS has a high spatial resolution compared to the other mass analyzers and has been used to image metals in many different tissues and cells.^46^ SIMS has been utilized to determine Pb and cadmium distribution in tree rings, cabbage leaves, and radish roots.^47–50^

SIMS is generally a nondestructive technique and has high sensitivity in the ppb range. However, it cannot penetrate deeply into a sample and is relegated to only the surface. Furthermore, the sample must be held in a high-pressure vacuum. SIMS can not only image trace metals, but biomolecules/interactions as well.^51,52^ This may be a preferred technique if looking for Pb bound within certain proteins. There have been recent advancements in SIMS, especially as it relates to the life sciences. These advancements include preparation techniques for improved subcellular imaging, as well as improved cluster beams for increased ion yields.^53,54^ While these advancements improve sensitivity for visualizing low concentrations of Pb, they also allow for parallel investigation of Pb-altered cell-to-cell interactions and metabolic responses. For an in-depth review of SIMS, see *Lockyer, 2014*.^55^

#### Matrix-Assisted Laser Desorption/Ionization (MALDI)

MALDI was originally coined in 1985. MALDI works by vaporizing and ionizing a sample with a laser. MALDI is paired with a mass spectrometer, most commonly a time-of-flight MS (MALDI-TOF). After ionization with MALDI, the ions enter a vacuum tube and are accelerated, thereby separating them. Depending on the size of the ions, they will arrive at the sensor at different times; smaller ions reach the sensor first, and heavier ones will lag. This allows for the precise determination of analyte spectra. Most commonly, MALDI-TOF is used in microbiology, especially for identifying microbes, from bacteria to fungus.^56–59^ In general, MALDI-TOF is useful for determining mixtures of complex proteins, by revealing individual protein sequences, molecular weights, etc.

While MALDI-TOF is not uncommon research infrastructure in the US, it does have some limitations, especially regarding detecting Pb or other metal ions. MALDI is a “soft ionization” technique, in that it transfers less energy to the sample so as not to cause fragmentation of proteins. Certain metal ions, like Pb, might not efficiently ionize from this. Furthermore, MALDI-TOF is based on (i.e., requires) a matrix that can absorb the energy from the laser to generate ions.^60^ Unlike ICP-MS, MALDI-TOF cannot detect metal ions in a solution, or free metal ions in a sample. However, it is a useful technique for determining Pb ions bound to protein complexes or other biomolecules. For instance, MALDI has been used to examine Pb­(II)-cystine complexes.^61^ MALDI has also been used to study other metals, such as copper bound to Alzheimer’s tau peptide.^62^ While MALDI can detect Pb as it interacts with biomolecules, sufficiently visualizing free Pb ions in biological samples can be difficult due to MALDI’s soft-ionization. However, recent advancements in MALDI have led to increased ion yields as well as improved resolution.^63,64^ With these advancements, MALDI can potentially be used not only to spatially visualize Pb in biological samples at high resolution, but also to simultaneously investigate biomolecule interactions. Furthermore, MALDI’s unique ability to identify microbes could allow for spatial visualization of Pb within the gut while concurrently determining which microbes are most affected by Pb exposure.

#### Glow Discharge Mass Spectrometry (GDMS)

GDMS is one of the oldest forms of MS, with glow discharge first incorporated in the 1920s as a means of producing ions.^65^ Glow discharge is a type of plasma that is generated by passing an electrical current through a gas. Argon ions are generated and then accelerated at the sample. Like SIMS, the sample is sputtered by the beam. Generated neutral species are ionized by the plasma and then transferred to the mass spectrometer to be sorted.^66^ Predominantly, GDMS has been used to analyze various samples in the material sciences, such as semiconductors, polymers, bulk metal, and ceramics.^67^ In regards to the life sciences, GDMS has been used to image Pb and other toxic heavy metals in biological tissues and samples, such as bone, blood, and urine.^68,69^ While GDMS is beneficial for quantifying Pb in solutions, it may be difficult to spatially visualize Pb in biological tissue. Generally, the output of GDMS is a spectrogram, like ICP-MS, but GDMS can be used to spatially visualize metals within samples by creating an “elemental map”.^70,71^ However, this is not standard and requires modification of the base instrument. Furthermore, GDMS is not suitable for high sample throughput and the sample must be conductive, or made to be conductive.^72,73^ Regardless of these potential difficulties in working with tissue samples, GDMS has seen some recent advancements in the form of improved metal detection and ionization capability, making it a potential attractive option for visualizing Pb.^74,75^

### X-ray Techniques

#### X-ray Fluorescence (XRF)

Originally devised in 1928, and not commercially available until the 1950s, XRF is a technique that utilizes X-rays to irradiate a sample. The atoms in the sample are excited and emit a specific energy, measurable via the XRF analyzer. The output is an X-ray spectrogram, allowing the user to determine the elemental components of the sample. The direct measurement of Pb ions in bone did not get under development until the 1970s (Figure 2), with its first published *in vivo* use in 1975.^76^ It was not until 1979 that the first study specifically looking at Pb in bones utilizing XRF was published (Figure 2).^77^

Unlike LA-ICP-MS, XRF does not destroy the sample in the process (Table 1). However, like LA-ICP-MS, XRF is not sensitive enough to measure pico or nano molar concentrations of elements and ions. XRF is most often found as a benchtop device, typically used for detecting ions within minerals. For detecting low-level concentrations within tissue, synchrotron X-ray microscopy (XFM) must be used. XFM is far more sensitive and has quicker analysis times than benchtop versions.^78^ XFM also allows for spatial information, unlike benchtop XRF which will only give the user a spectrogram. XFM has been used to spatially visualize Pb and other metals in a variety of tissues, including mouse brains, leeks, and Chinese cabbage leaves.^79–82^ While XFM is one of most common techniques for spatially visualizing Pb in biological samples, it is an expensive and complicated technique, which greatly narrows its use. XFM is not common among research infrastructure within the US. However, XRF and XFM have seen some advancements in recent years, such as metal-based probes for use with XFM, potentially allowing a unique way to visualize Pb trafficking within cells.^83^ Further advancements have been made to increase the sensitivity of portable XRF, allowing flexible use of XRF for life scientists in the field or at the bench, as well as furthering accessibility in terms of affordability and simplicity.^84^ For a more in-depth review of XRF, see *Pushie et al, 2022*.^85^

#### Particle-Induced X-ray Emission (PIXE)

PIXE was first proposed in 1970 and then developed during the 1970s. PIXE works by accelerating protons in a particle accelerator and then firing that beam onto a sample. The concentrated proton energy causes electrons in the sample to undergo inner-shell ionization, and the outer-shell electrons drop to the inner shells, giving off a specific X-ray energy spectrum. Although PIXE is not as common today as XFM or LA-ICP-MS, PIXE was one of the first methods used to spatially analyze Pb in biological samples (Figure 2).^86–88^ More recently, PIXE has been used to analyze iron in the rabbit cerebellum,^89^ Pb in vegetables,^90^ and Pb in cancerous and noncancerous human breast tissue.^91^

PIXE is similar to XRF, in that they both measure X-ray spectra. However, XRF uses direct X-ray excitement of the sample, rather than accelerated protons. The fact that PIXE requires a particle accelerator is a drawback, since this is more expensive than an XRF or XFM setup (i.e., synchrotron facility). Furthermore, unlike XFM, PIXE cannot penetrate deeply into a sample, and it is generally restrained to the first upper micrometer (Table 1). However, PIXE is more sensitive than benchtop XRF and can potentially be even more sensitive than XFM, depending on calibration. Similar to XFM, PIXE is nondestructive. PIXE can also be used to scan micrometer dimensions, a technique called micro-PIXE. Micro-PIXE can generate images for spatial detection of elements and ions, along with a spectrogram, just like LA-ICP-MS and XFM. Due to the specific caveats, PIXE is not as commonly utilized as XRF or LA-ICP-MS but still is a consideration for scientists who need sensitive spatial detection of metal ions in tissue. 50 years after the development of PIXE, technological advancements continue to emerge. Some of these recent advancements include improvements in metalloprotein analysis, as well as incorporating machine-learning models to aid in elemental analysis and classification.^92,93^ These advancements can be beneficial in understanding how Pb interacts with biomolecules, and PIXE-associated machine-learning can help classify or determine at-risk organs and cell-types.

### Other X-ray-Based Techniques

#### Microtomography (Micro-CT)

Micro-CT is a nondestructive imaging technique that utilizes X-rays to produce 3D images from many 2D slices. As the X-ray passes through the sample, the intensity changes based on the distance, sample material, and source energy. This attenuation is used to determine the density of tissue as the reduced intensity beams are collected by a detector. Unlike many of the techniques discussed in this paper, micro-CT can be used both *in vitro* and *in vivo.* This allows for potential analysis in a living animal. Micro-CT has generally been used to determine bone structure, especially in diseases like osteoarthritis. However, in the past two decades, micro-CT has been used successfully to image metals within plant tissue.^94^ Micro-CT has recently been used to spatially visualize cadmium within fish tissue.^95^ Micro-CT offers a unique and interesting way to determine spatial distribution of Pb within tissue. It also has potential to view Pb distribution *in vivo*, without the need for dissection or sample preparation, allowing for longitudinal assessments within the same organism. However, micro-CT is specialized, expensive equipment with potential limitations in sensitivity below micromolar concentrations of Pb.^96^ Some recent advancements in Micro-CT include increased accuracy in multiorgan segmentation as well as improved visualization of micron-scale cellular structures.^97,98^ These advancements may aid in our understanding of how Pb is spatially distributed within organs on the systemic, cellular, and organellar levels.

#### X-ray Photoelectron Spectroscopy (XPS)

XPS is another X-ray-based imaging technique that utilizes the photoelectric effect (i.e., the emission of electrons). The X-rays directed at the sample generate photoelectrons which then enter a vacuum. From here, the photoelectrons are detected by an electron detector system and then analyzed using, most commonly, a hemispherical electron analyzer. XPS is generally relegated to just the upper-most nanometers of a sample,^99^ giving it much less depth than most other techniques, and sensitivity usually falls into the PPM range. However, lower limits can potentially be achieved.

XPS is often utilized to examine metals on the surface of crystals,^100^ medical devices,^101,102^ and metal complexes and clusters.^103,104^ In biological sciences, XPS is often used to determine biochemical properties of a sample, such as proteins, amino acids, and saccharide composition. XPS has been used to determine surface protein and amino acid composition in fungus,^105^ and surface chemical composition of rice and flour.^106^ XPS has been used to analyze metals in samples, such as this study which looked at Pb­(II) reduction in bacteria.^107^ While XPS has shallower depth-limits than other discussed techniques, some recent advancements have been made to increase potential depth.^108^ These advancements make XPS a useful technique for those interested in spatially visualizing Pb in thin cross sections of biological samples.

## Discussion

The past 50 years have seen a surge in techniques employed to quantify and visualize Pb and other metals within biological tissues. Many of these techniques utilize X-rays or mass spectrometry to excite metal ions. Each technique offers certain benefits and caveats that must be weighed carefully when determining which approach is best suited for a scientist’s specific needs. Some techniques, such as LA-ICP-QQQ, have fantastic sensitivity but will destroy the sample in the process. Other methods, like XFM, keep the sample intact but can only scan a small amount of the sample. Other methods, like MALDI-TOF, cannot view free Pb ions in solution but can view them if they are bound to biomolecules like proteins. Furthermore, some of these techniques may be limited to researchers due to access or cost. An investment and development of suitable benchtop/portable methods to detect Pb in reliable, economical, and standardized manners will not only have a substantial impact on scientific accessibility and rigor but will also be suitable for a vast number of model organisms and approaches in the life sciences. Therefore, it is important to not only consider the compatibility of Pb detection techniques affording spatial and quantitative information with gold standard assays in respective life science fields, but also to consider affordability and simplicity when designing future probes.

Outside of the methods discussed, there are less explored techniques that may offer novel approaches to spatially visualize Pb. For example, PET scans are widely used in medicine to measure a variety of physiological functions, including blood flow and absorption.^109,110^ PET scans require radiolabeled tracers, since they rely on β plus decay for imaging. During β plus decay, a positron is emitted and interacts with an electron, emitting γ rays. PET scans are highly sensitive and can detect most radiotracers in the picomolar range. PET scanners are common at hospitals and medical research facilities. However, PET scanners themselves are expensive to purchase, house, service, and operate. In addition, the use of radiolabeled tracers can present some problems when imaging tissue. Working with radiolabeled tracers is dangerous and much care must be taken when handling, especially when working with animal models. Furthermore, the half-life of many radiotracers is short. Pb-212, for instance, has been used as a diagnostic isotope.^111^ Its half-life is only 10.6 h. Since radioactive decay is exponential, PET cannot detect Pb for extended periods of time.^111^ While conducting short-term studies is feasible, this short half-life makes conducting long-term longitudinal studies (i.e., more than 7 days) more difficult. While PET scans have been used with Pb as a tracer *in vivo* in mice to visualize tumor uptake, it has not been used to analyze the spatial distribution of Pb in healthy animals.^112,113^ While PET scans offer a unique and currently underutilized technique to spatially visualize Pb, they still are limited to researchers due to access and cost. Advances in the synthesis and development of fluorescent tracers and probes may offer new avenues to overcome these complications.

Fluorescent dyes and probes used for detecting endogenous metals within cells are common in the world of biology. For instance, some traditional probes, such as *fura-2* and *indo-1*, can sensitively measure intracellular calcium ion flux.^114,115^ Originally designed for calcium imaging, fura-2 does indeed have a strong affinity for calcium ions; it is highly sensitive and is able to detect calcium in the nanomolar range. However, it also has affinity for other endogenous metal ions such as copper, iron, manganese, and zinc, as well as an affinity to heavy metal toxicants such as cadmium, nickel, and barium.^116^ Pb is no exception; both fura-2 and indo-1 can detect Pb intracellularly and can be utilized for this purpose^117,118^ The lack of specificity with these probes results in challenging confounds when attempting to measure Pb with precision, especially since the fluorescent output of Pb is similar to what one would expect from calcium or zinc ions.^116^

In the past two decades, some Pb-specific fluorophores and optical chemical sensors have been developed (Figure 2), which allow for more precise visualization of Pb ions within living cells.^119–121^ However, due to the many shared intrinsic chemical and physical properties of transition and post-transition metals, designing fluorophores that are uniquely specific to Pb is difficult. For example, one Pb-specific fluorophore, Leadmium Green AM, has mainly been used to visualize cadmium in plant tissue and cells *in vitro*, possibly owing to its higher affinity to cadmium than Pb itself.^122–125^ Optical chemical sensors such as *JA/(2,6-di­((E)-benzylidene)­cyclohexan-1-one)* (JA) form a complex with Pb ions and fluorescence can be detected via simple spectroscopic techniques.^126^ Importantly, this sensor showed limited interference from endogenous metals like sodium and zinc, as well as toxic heavy metals like cadmium. Another chemical fluorescent sensor, EABH, stops fluorescing when bound to Pb ions, but continues to fluoresce in the presence of other metal ions.^127^

Such aforementioned probes solve a few different problems with current techniques used to spatially detect Pb. First, they are simple and affordable. They can either be bought as a stock or synthesized with only a few inexpensive reagents. Their application is straightforward, and the equipment needed is not complicated to use. For example, for the JA optical sensor, only a benchtop UV–vis spectrometer is required. Second, the throughput is high, due to the simplicity of sample preparation and fluorophore application. However, most of the probes require living cells and cannot be used in fixed tissue. It may be possible to visualize Pb fluorescence after fixation, if cells uptake fluorophore before fixation and the fluorescence is not quenched. Furthermore, a probe could be designed for use *in vivo* to act as a “tracer”, like in PET, but avoiding the associated radiolabeled tracers.

While these probes could be designed as *in vivo* tracers to spatially visualize Pb, another interesting and immediate use case for these probes involve organoids and organs-on-a-chip (OOC). Organoids are simplified 3D versions of an organ grown in a dish and often even mimic some of the organ-specific basic functions. OOCs are essentially multiple different organoids on a microfluidic chip, where each organoid can interact with another in an isolated system. Exposing an organoid to Pb and then applying a Pb-specific probe may grant insight not only into the spatial distribution of Pb within an organoid, but also intercellular trafficking and resorption dynamics in real-time. OOCs can take this a step further, by revealing how Pb is transferred between various organoids and how Pb spatially distributes between and within them. Furthermore, while total subcellular and organellar Pb content has been studied, these probes could help in our understanding of how Pb is physically trafficked subcellularly between organelles that vary in calcium activity.^128^ Utilizing these probes not only to elucidate how Pb is spatially dispersed within tissue, but also how Pb is trafficked from organ to organ, cell to cell, and even between organelles, will help guide the development of novel cell-based therapies and regenerative engineering research.

Developing novel therapies on both the systemic and cellular level will require understanding correlations between Pb levels in tissue and BLLs. However, even with current data, it is difficult to determine exactly which populations are most at risk of Pb exposure. Some communities are still actively inventorying Pb lines, and there are many variables involved in determining total Pb exposure (e.g., galvanized Pb pipe or just Pb goosenecks, length of pipe, how the water itself is sampled, etc.).^129^ Furthermore, even though drinking water is the main route of Pb exposure in the US, there are many other sources, as discussed earlier, that are harder to track. Routine and mass blood tests, which were conducted in the US in the 1960s, may be a solution, but these measurements are still a transient measure of Pb exposure.^130^ Capitalizing on routine biopsies or discarded tissue (e.g., cysts, hair transplants, etc.) may expedite filling this gap, since each organ uptakes Pb in different ways, depending on route of exposure, duration of exposure, and age. As we move toward cell-based approaches in the toxicological and biological sciences, it will be important to have a baseline understanding of how toxicants impact the body on both a systemic and cellular/subcellular level to foster bidirectionality between informative *in vitro* and *in vivo* models.
